# *Salmonella* Typhimurium Strain ATCC14028 Requires H_2_-Hydrogenases for Growth in the Gut, but Not at Systemic Sites

**DOI:** 10.1371/journal.pone.0110187

**Published:** 2014-10-10

**Authors:** Lisa Maier, Manja Barthel, Bärbel Stecher, Robert J. Maier, John S. Gunn, Wolf-Dietrich Hardt

**Affiliations:** 1 Institute of Microbiology, ETH Zürich, Zurich, Switzerland; 2 Max von Pettenkofer-Institut, München, Germany; 3 German Center for Infection Research (DZIF), partner site LMU Munich, Munich, Germany; 4 Department of Microbiology, University of Georgia, Athens, Georgia, United States of America; 5 Department of Microbial Infection and Immunity, Center for Microbial Interface Biology, Biomedical Research Tower, The Ohio State University, Columbus, Ohio, United States of America; Charité-University Medicine Berlin, Germany

## Abstract

*Salmonella enterica* is a common cause of diarrhea. For eliciting disease, the pathogen has to colonize the gut lumen, a site colonized by the microbiota. This process/initial stage is incompletely understood. Recent work established that one particular strain, *Salmonella enterica* subspecies 1 serovar Typhimurium strain SL1344, employs the *hyb* H_2_-hydrogenase for consuming microbiota-derived H_2_ to support gut luminal pathogen growth: Protons from the H_2_-splitting reaction contribute to the proton gradient across the outer bacterial membrane which can be harvested for ATP production or for import of carbon sources. However, it remained unclear, if other *Salmonella* strains would use the same strategy. In particular, earlier work had left unanswered if strain ATCC14028 might use H_2_ for growth at systemic sites. To clarify the role of the hydrogenases, it seems important to establish if H_2_ is used at systemic sites or in the gut and if *Salmonella* strains may differ with respect to the host sites where they require H_2_ in vivo. In order to resolve this, we constructed a strain lacking all three H_2_-hydrogenases of ATCC14028 (14028^hyd3^) and performed competitive infection experiments. Upon intragastric inoculation, 14028^hyd3^ was present at 100-fold lower numbers than 14028^WT^ in the stool and at systemic sites. In contrast, i.v. inoculation led to equivalent systemic loads of 14028^hyd3^ and the wild type strain. However, the pathogen population spreading to the gut lumen featured again up to 100-fold attenuation of 14028^hyd3^. Therefore, ATCC14028 requires H_2_-hydrogenases for growth in the gut lumen and not at systemic sites. This extends previous work on ATCC14028 and supports the notion that H_2_-utilization might be a general feature of *S.* Typhimurium gut colonization.

## Introduction

The gut lumen is colonized by a dense microbial community called the microbiota. The microbiota performs numerous important functions which have been the topic of intense recent research (reviewed in [1]). One prominent function is the consumption of complex carbohydrates which the host is not able to digest. This is facilitated by primary fermenters which break down dietary and mucus-derived polymers and ferment the monomers into short chain fatty acids, lactate, CO_2_, formate and H_2_ [2]. These primary fermentation products are subsequently absorbed by the host, consumed by secondary fermenters or released into the atmosphere. Importantly, the metabolic activity of the microbiota limits gut luminal nutrient availability for incoming bacteria and thereby helps to prevent infection (“colonization resistance”; [2]–[4]). Enteric pathogens must have the ability to overcome colonization resistance in order to cause infection. However, these strategies are still not well understood.

*Salmonella enterica* is a Gram-negative bacterial species eliciting enteric infections in a wide range of hosts [5], [6]. In warm-blooded animals, most infections are caused by *S. enterica* subspecies 1, e.g. serovar Typhimurium. Using the *S*. Typhimurium strain SL1344, we have recently begun to investigate how the pathogen can establish in the host's gut in the face of an intact microbial community [7]–[9]. In this initial phase of colonization, the mucosa does not yet show any overt symptoms of disease and microbiota metabolism is thought to function normally. Here, SL1344 was found to capitalize on molecular hydrogen (H_2_), a central product of microbiota metabolism [8]. Specifically, H_2_ serves as an electron donor consumed by H_2_-hydrogenases, i.e. the *hyb*-hydrogenase. This is a well-characterized cytoplasmic membrane enzyme complex which abstracts the electrons from H_2_ and channels them into the ubiquinone pool [10]–[17]. During SL1344 growth in the mouse gut, about 90% of these electrons are transferred to fumarate, a step catalyzed by the fumarate reductase (*frd*; [8]). Overall, this anaerobic H_2_-consumption fuels SL1344 growth to such an extent that hydrogenase mutants are 100-fold attenuated in competitive gut colonization assays. This is true for the *hyb* mutant of SL1344 and for a SL1344 mutants lacking all three H_2_-hydrogenases. However, it had remained unclear, if this also holds for other *Salmonella* strains.

In many cases, mechanisms discovered in one strain are equally relevant for other strains of the serovar Typhimurium and often even for the entire *S. enterica* species. However, there is accumulating evidence that this is not always the case. Strain-specific differences in virulence, growth or other phenotypes can arise from sequence variations or differences in gene content (see below). While, *S*. *enterica* strains can differ by as much as 65 to 99% of their genetic content [18]–[22], many strains from the serovar Typhimurium are much more similar to each other [23], [24]. The *S*. Typhimurium strain ATCC14028 employed in this study differs from strain SL1344 by just 2.6% of its genome [24], [25]. These differences comprise the prohage SopEΦ (present in SL1344 [26], [27], not ATCC14028), the prophage Gifsy-3 (present in ATCC14028, not SL1344; [28]), different plasmid contents, a histidine auxotrophy (in SL1344, not ATCC14028) [29], as well as numerous sequence polymorphisms distributed throughout the genomes (e.g. one T→C change in a H_2_-hydrogenase operon, resulting in an R188→G188 amino acid exchange in HyaB2). In many cases (including the H_2_-hydrogenase operons), the functional consequences of the presence, the absence or the mutation of a particular gene have remained unclear. SopEΦ is a notable exception. This prophage encodes a gene cassette (“moron”) in its tail-fiber region which encodes SopE [30]–[32], a RhoGTPase activating effector protein which is injected into host cells via the SPI-1 type III secretion system [33], [34]. SopE dramatically enhances the capacity of *S*. Typhimurium strains to trigger membrane ruffling and elicit mucosal infection in cows and mice [33]–[36] Moreover, the absence of SopE (or SopEΦ) was found to explain why ATCC14028 (but not SL1344) utilizes the terminal electron acceptor tetrathionate for anaerobic respiration in the lumen of the inflamed gut [37]. This was of particular interest, as both strains encode for the genes required for anaerobic tetrathionate utilization. Thus, genetic comparison alone seems insufficient to predict the utilization of metabolic pathways *in vivo*, as genetic differences in unrelated genes (e.g. the virulence factor SopE) can substantially affect metabolic preferences in complex environments such as the mouse intestine. Therefore, experimental verification is indispensable to address the question whether a particular anaerobic pathway is used by a given *Salmonella* strain.

Indeed, earlier work on *S*. Typhimurium strain ATCC14028 suggested that differences in H_2_ metabolism might exist [14]. H_2_-hydrogenase mutants of this strain were found to be strongly attenuated at colonizing systemic sites. This was taken as evidence that ATCC14028 uses H_2_ to fuel growth, but it had remained unclear if this was attributable to H_2_-dependent growth in these organs or in the intestinal tract. In fact, this H_2_-fuelled growth of ATCC14028 at systemic sites seemed plausible, as microbiota-derived H_2_ is well known to diffuse even to distant sites in the body (an average of 40 µM of microbiota-derived H_2_ are found in the mouse liver/spleen [14]) and significant amounts of H_2_ are exhaled via the lungs [38], [39]. This left us with the possibility that different *S*. Typhimurium strains may use microbiota-derived H_2_ at different sites i.e. the gut lumen (strain SL1344) or at systemic organs (strain ATCC14028). However, it could not be excluded, that this was simply attributable to slight differences in the experimental design and the subsequent interpretation of the data. It is important to note that the ATCC14028 experiments had been performed in the typhoid fever model of *Salmonella* infection [14], [40]. In this type of experiment, the mice are inoculated via the oral route and the pathogen traverses the intestinal mucosa before disseminating to systemic sites. This left room for an alternative interpretation of the ATCC14028 data: the systemic colonization defect of ATCC14028 hydrogenase mutants might be attributable to a brief phase of gut luminal pathogen growth. A gut luminal growth defect of the ATCC14028 hydrogenase mutant could have skewed the ratio of wild type vs mutant bacteria before systemic colonization was initiated. However, gut luminal growth had not been monitored in the previous study, and it remained unresolved if H_2_-fuelled growth in the gut lumen may have contributed to the phenotype. Therefore, it remained to be established whether ATCC14028 uses microbiota-derived H_2_ for colonizing the gut lumen, or for growth at systemic sites.

## Results and Discussion

### H_2_-hydrogenases are required for efficient gut colonization by ATCC14028

ATCC14028 is known to encode three H_2_-hydrogenases which are largely identical to the operons in SL1344. In order to generate an isogenic H_2_-hydrogenase deficient mutant, we disrupted all three H_2_-hydrogenases (14028^hyd3^; Materials and Methods). For studying gut colonization in the face of an intact microbiota, we employed the LCM model. LCM mice are ex-germfree C57BL/6 mice which had been colonized by the 8 strains of the altered Schädler flora and which had incorporated several dozen of additional strains into their microbiota during subsequent housing [8], [9]. Importantly, the microbiota of LCM mice features most characteristics of a typical complex microbiota, including phylum-level composition, microbiota cell density and the ability to generate a steady state level of about 50 µM H_2_ in the cecum lumen [7]–[9], [14]. Importantly, these mice do feature an attenuated colonization resistance. This is quite different from mice with a complex, specified pathogen-free (SPF) microbiota (further termed SPF), which allow only low-level gut colonization by *Salmonella* spp. in most mice (approx. 10^2^–10^6^ cfu/g in 95% of the animals tested; [9], [41], [42]. Thus, efficient and reproducible gut colonization of SPF mice by *S*. Typhimurium is only achieved upon antibiotic treatment which transiently disrupts the microbiota and alleviates colonization resistance [41], [43]–[50]. In LCM mice, *S*. Typhimurium SL1344 can grow up in the gut lumen and reaches colonization densities of 10^8^ cfu/g by day 1 p.i., reaches 10^9^ cfu/g by day 3 and gut inflammation is triggered around day 3 p.i. [8]. Therefore, the LCM mice allow studying how *S*. Typhimurium establishes gut luminal colonization in the face of an intact microbiota.

**Figure 1:**
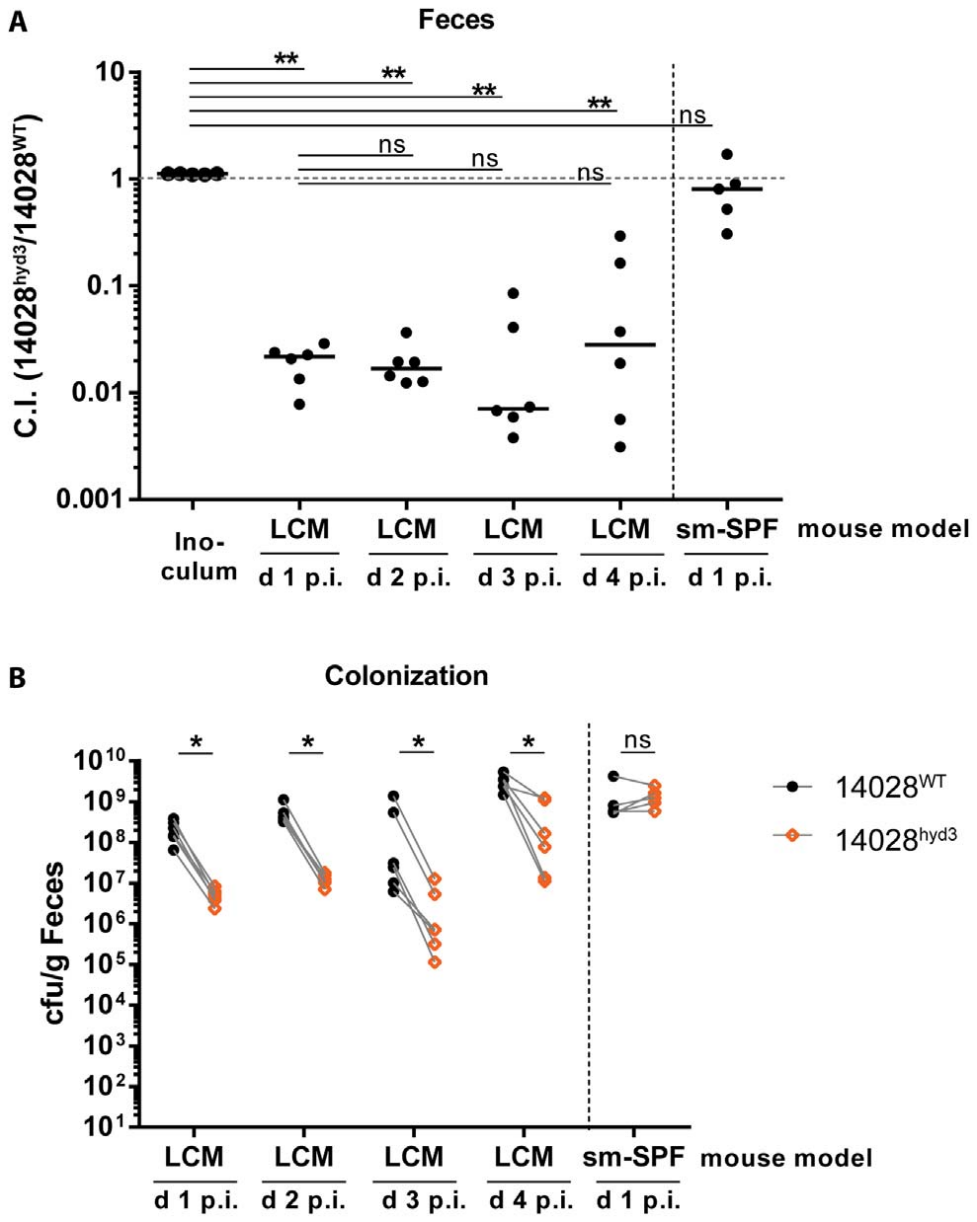
14028^hyd3^ is impaired in early gut ecosystem invasion. Gut colonization was monitored in two different mouse models: streptomycin-pretreated conventional mice (sm-SPF) and low complexity microbiota (LCM) mice. Mice were infected with a 1∶1 mixture (5×10^7^ cfu by gavage) of 14028^hyd3^ and the isogenic background strain (14028^WT^). Fecal loads of both strains were determined by selective plating. (A) Competitive infection indices were determined over 4 days. Ns  =  not significant (P≥0.05), ** P<0.01, Mann-Whitney U test. (B) Bacterial loads of both competing strains (14028^WT^ and 14028^hyd3^) are depicted Ns  =  not significant (P≥0.05), * P<0.05; one-tailed Wilcoxon matched pairs signed rank test on paired data (dashed lines).

**Figure 2:**
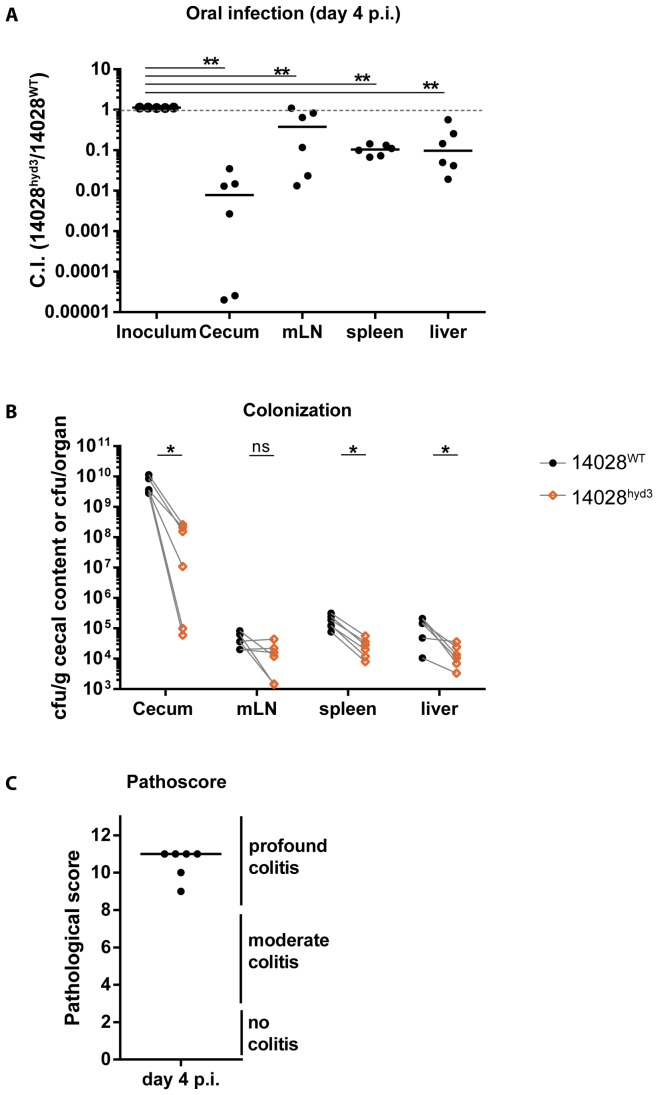
Oral infection experiments revealed that in ATCC14028, hydrogenases fuel pathogen growth in the intestine. (A) LCM mice from Figure 1 were sacrificed at day 4 post infection and competitive indices in the cecum and at systemic sites were determined. ** P<0.01, Mann-Whitney U test. (B) Bacterial loads in the cecum and at systemic sites of both competing strains are plotted. Ns  =  not significant (P≥0.05), * P<0.05; one-tailed Wilcoxon matched pairs signed rank test on paired data (dashed lines). (C) Cecal tissue sections were HE-stained and scored for intestinal inflammation.

LCM-mice were infected with a 1∶1 mixture of wild type ATCC14028 (14028^WT^) and 14028^hyd3^ via the oral route (5×10^7^ cfu in total, by gavage). We analyzed the bacterial loads in the feces at days 1–4 p.i. (Fig. 1A, B), monitored pathogen loads in the cecum lumen, the mesenteric lymph nodes, the spleens and the livers, and analyzed the mucosal inflammation at day 4 p.i. (Fig. 2A–C). In the feces of the LCM-mice, 14028^hyd3^ featured a pronounced colonization defect already by day 1 p.i. (competitive index C.I. 0.02; Fig. 1A, B). During the subsequent three days, the total fecal pathogen loads rose from ≈10^8^ cfu/g to about 10^9^ cfu/g while the C.I. did not drop any further. Control infections were performed in streptomycin pretreated conventional mice (5×10^7^ cfu in total, by gavage; 1 day infection). In these animals, the microbiota is transiently disrupted by streptomycin and 14028^hyd3^ does not feature any gut luminal colonization defect (Fig. 1A, B). These data are strikingly similar to our earlier data obtained with H_2_-hydrogenase mutants of SL1344 [8] and indicated that ATCC14028 can subvert H_2_ for gut luminal colonization.

In the cecum lumen, 14028^hyd3^ had a similar colonization defect as in the feces (Fig. 2A, B) and all mice featured pronounced mucosal inflammation by day 4 p.i. (Fig. 2C). Furthermore, we detected a significant colonization defect of 14028^hyd3^ in the mLN, the spleens and the livers of the LCM mice (Fig. 2A, B). However the attenuation appeared to be slightly less pronounced than in the cecum lumen and in the feces. However, these data could not unequivocally settle whether H_2_-hydrogenase dependent growth might contribute to some extent to systemic colonization.

### H_2_-hydrogenases do not contribute to systemic growth of ATCC14028

**Figure 3:**
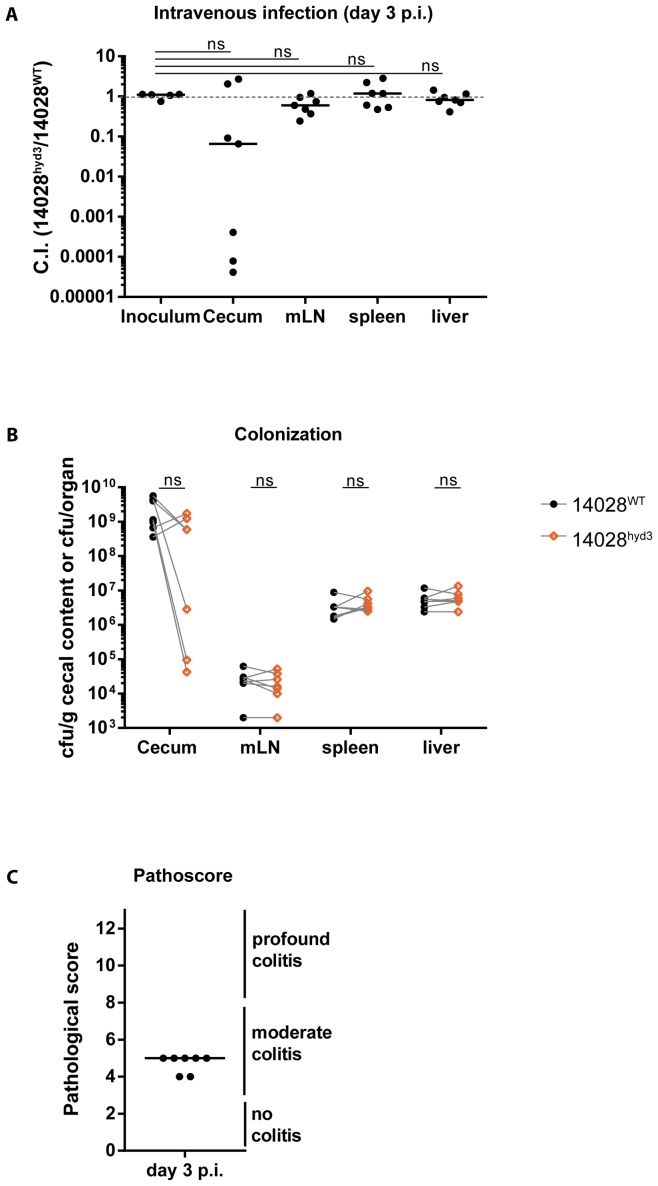
Intravenous infection experiments verified that in ATCC14028, hydrogenases are not required for growth at systemic sites. (A) LCM mice were intravenously infected with a 1∶1 mixture of the 14028^hyd3^ and the isogenic background strain (14028^WT^) (5×10^3^ cfu). Animals were sacrificed at day 3 p.i. and competitive indices in the cecum and at systemic sites were determined. Ns  =  not significant (P≥0.05), Mann-Whitney U test. (B) Bacterial loads in the cecum and at systemic sites of both competing strains are plotted. Ns  =  not significant (P≥0.05); one-tailed Wilcoxon matched pairs signed rank test on paired data (dashed lines). (C) Cecal tissue sections were HE-stained and scored for intestinal inflammation.

In a second approach, we specifically addressed whether H_2_-hydrogenases contribute to systemic colonization. To this end, we infected LCM-mice via the intravenous route with a 1∶1 mixture of 14028^WT^ and 14028^hyd3^ (5×10^3^ cfu in total, i.v.). After three days, the animals were sacrificed and we analyzed the pathogen loads (and the C.I.) in the cecum lumen, the mLN, the spleens and the livers and assessed gut inflammation in the cecum tissue (Fig. 3A–C). The total pathogen loads in the mLN (≈10^4^–10^5^ cfu), the spleens (≈10^7^ cfu) and the livers (≈10^7^ cfu) were well in line with published data for i.v. infections in C57BL/6 mice [51]. Strikingly, 14028^hyd3^ did not feature any detectable colonization defect in the systemic organs after i.v. infection (p≥0.05; C.I.≈1; Fig. 3A). Colonization defects of 14028^hyd3^ were only detected in the cecum lumen in 5 out of 7 mice. This population must have arisen by pathogen dissemination from systemic sites to the gut lumen, e.g. by pathogen routing via the gall bladder, by phagocyte-mediated transport to the gut tissue [52]–[55] or by oral ingestion by licking the injection site at the tail. In any case, our data suggest that the growth defect of 14028^hyd3^ has most likely arisen after the pathogen had arrived in the gut lumen.

These data established that ATCC14028 does not require H_2_-hydrogenases for growth at systemic sites if the gut is bypassed during the infection procedure. In the typhoid fever model [14] or oral infections of LCM mice, gut luminal growth of the bacteria seems to precede the spread to systemic sites. This gut luminal growth most likely explains why H_2_-hydrogenase mutants are found in lower numbers in the mLN, livers and spleens of the animals than the isogenic wild type strain.

It should be noted that the 14028^hyd3^ mutant used in our study lacked all the three uptake-type H_2_-hydrogenases. Thus, formally we cannot rule out that the requirement of a single hydrogenase is masked by the absence of the other two hydrogenases. For example, deletion of one hydrogenase might increase *S*. Tm fitness, while deletion of another hydrogenase might decrease *S*. Tm fitness. By analyzing both deletions in combination, the two opposed effects will be compensated. However, this seems unlikely, as none of the H_2_-hydrogenase mutants of SL1344 or ATCC14028 that have been analyzed in the past had featured higher virulence than the isogenic wild type strain [8], [14]. Nevertheless, mutants lacking just one of the H_2_-hydrogenases at a time would have to be studied in detail to address this in a systematic fashion. In addition, differential expression of the three H_2_-hydrogenases [15], [17], strain-specific differences in the expression patterns and microbiota/environment-specific cues (e.g. different H_2_ availability) might play a role. Indeed, the three different hydrogenases have different hydrogenase activities [16]. Moreover, in typhoid fever model infections of SPF mice, ATCC14028 may utilize several different H_2_-hydrogenases [14]. In contrast, SL1344 growth in the gut lumen of LCM mice relied exclusively on *hyb*, not the other H_2_-hydrogenases [8]. The environmental cues steering the differential hydrogenase expression in vivo remain to be established. Nevertheless, it seems quite safe to assume that the gut lumen is the site where H_2_-utilization by *S*. Typhimurium is most prominent. Still, H_2_ could represent an auxiliary reductant for *Salmonella* at systemic sites under otherwise poor nutrient conditions, or when the microbiota is especially active in fermentative metabolism (e. g. high H_2_ production).

In conclusion, our data establish that ATCC14028 is strikingly similar to SL1344 in requiring H_2_-hydrogenases for growth in the gut, not at systemic sites. This may suggest that the use of H_2_ for gut luminal colonization is a general feature of *Salmonella* Typhimurium strains.

## Materials and Methods

### Bacterial strains

All strains used in this study are derivates of the *Salmonella enterica* serovar Typhimurium ATCC14028 (IR715), in which a streptomycin resistance was added by P22 phage transduction of the *aadA* gene from *S*. Tm SL1344 [35]. Deletions in the hydrogenase genes were constructed by lambda/red homologous recombination [56] as described previously [8] (Table 1).

**Table 1:** Bacterial strains used in this study.

Strain	Genotype	Reference
14028^WT^	Streptomycin-resistant derivative of IR715 (constructed by P22-transduction of *aadA* gene from *S*. Tm SL1344 into the ATCC14028 derivative IR715)	[35]
14028^hyd3^	ΔSTM3147-3150, STM1786-87::*aphT*, STM1538-1539::*cat*	This study

### Mouse infection experiments

All mice used in this study are C57BL/6 background and bred at the Rodent Center HCI (RCHCI) (ETH Zurich, Switzerland). Low complex microbiota (LCM) mice are ex-germfree mice which were colonized with the Altered Schaedler flora-cocktail in 2007 [9] and ever since bred under strict hygienic isolation. Co-infection experiments were performed as described previously [41] in 8 to 10 week old mice. Pre-treatment with 20 mg streptomycin was only performed if indicated (Figure 1). For infection, both bacterial strain (14028^WT^ to 14028^hyd3^) were grown for 12 h in 0.3 M NaCl supplemented LB medium, diluted 1∶20 and sub-cultured for 4 h in the same medium and mixed in a 1∶1 ratio. For oral infections, mice were infected with 5·10^7^ cfu bacteria by gavage. For intravenous infections, 5·10^3^ cfu bacteria were injected into the tail vein. Mice were sacrificed on day 1 p.i., day 3 p.i or day 4 p.i. by cervical dislocation. Freshly collected fecal pellets, cecum content and organs were homogenized in PBS (0.5% tergitol, 0.5% bovine serum albumin). Differential plating on MacConkey agar (Oxoid) supplemented with the appropriate antibiotics (50 µg/mL streptomycin, 50 µg/mL kanamycin and 30 µg/mL chloramphenicol) was performed to determine bacterial population sizes. The competitive index was calculated by division of the population size of 14028^hyd3^ by the population size of 14028^WT^. This ratio as corrected for the ratio of both strains in the inoculum. Parts of the cecal tissue were embedded in OCT (Sakura), cryosections were prepared and stained with hematoxiline/eosine. HE-stained sections were evaluated by scoring for submucosal edema, PMN infiltration, presence of goblet cells and epithelial damage with a maximum score of 13 [57].

### Statistical analysis

The one-sided Wilcoxon matched-pairs signed rank test and the exact Mann-Whitney *U* test were performed using the software Graphpad Prism Version 6.0 for Windows (GraphPad Software, La Jolla California USA, www.graphpad.com). P values of less than 0.05 were considered as statistically significant. To compare C.I.s to C.I. of inoculi, ratios of 14028^hyd3^ and 14028^WT^ were compared to the ratio of both strains in the inoculum using an exact Mann-Whitney *U* test.

### Ethical statement

All animal experiments were reviewed and approved by the Kantonales Veterinäramt, Zürich (license 223/2010 & 222/2013) and are subject to the Swiss animal protection law (TschG).
